# Differential Analysis of Volumetric Strain Method Characterization in the Context of Phase Change of Water in Carbonate Rocks

**DOI:** 10.3390/ma15010308

**Published:** 2022-01-02

**Authors:** Karol Skowera, Zbigniew Rusin

**Affiliations:** Department of Organization of Construction and Building Materials, Faculty of Civil Engineering and Architecture, Kielce University of Technology, 25-314 Kielce, Poland; zrusin@tu.kielce.pl

**Keywords:** limestone, carbonate rock, differential analysis of volumetric strain, phase change

## Abstract

Modernized technological processes or increasing demands on building materials force the scientific community to analyze in more detail the suitability of individual raw materials and deposits. New or modernized research methodologies make it possible to better understand not only the geometrical structure of the pore space of materials but also the processes taking place in them and the interaction of many factors at the same time. Despite the extensive literature in the field of research on capillary-porous materials, scientists still face many challenges because not everything is known. Carbonate rocks are the most common (one-tenth of Earth’s crust) sedimentary rocks. Analysis of the test results obtained with the use of the modernized differentia analysis of volumetric strain (DAVS) methodology allows for a better adjustment of rock deposits to the products that can be produced from them. In this manner, it is possible that it will contribute to a more rational use of exhaustible rock deposits and not only carbonate ones. This research subject is of great importance for modern science, which was also noted in many of science publications.

## 1. Introduction

The pore space of capillary-porous materials is defined by an innumerable number of smaller or larger pores of different sizes, shapes, etc. We can distinguish at least two types of pores or voids appearing in the material. The first type includes pores that form an interconnected or, in other words, effective system. Pores open on one side only, and the so-called blind pores can also be included in this type of pores. The second type includes pores that are not interconnected with each other—isolated. It is obvious that these pores are not able to transport liquid inside the material space and, therefore, do not participate in this process [1,2].

Porosity is one of the simplest physical properties that allows the quality of a material to be determined. We distinguish between total porosity and the porosity that corresponds only to pores that are connected to each other. Total porosity refers to all voids in the material that are also not directly connected to each other and do not take part in liquid transport inside the material [3,4]. Normal porosity characterizes only a pore space that is interconnected with one another.

With respect to awareness of the importance of the function of the pore space in the material, researchers define it by using various methods. These include optical methods [5], nuclear magnetic resonance [6], mercury porosimetry [7] or even (indirectly) material absorbability [5]. Indirect methods also include methods involving gas adsorption/desorption, electrofusion and ultrasonic methods [8,9].

A method that allows indirect determination of pore size distribution in a capillary-porous material is also the differential analysis of volumetric strain—DAVS (dilatometric method). The modernized methodology allows determining the dimensions of the entrances to the pores during the cooling process of the sample and the internal dimensions of the pores on the basis of its heating. The method of differential analysis of volumetric strain with various modifications has been used in pore space studies for many years [10,11,12]. Its main task is the qualitative and quantitative assessment of the pores in the material in terms of the possibility of absorbing water, which is or is not able to freeze under operating conditions [13,14]. Over the years, the DAVS method has significantly evolved and been modernized. Currently, the DAVS method is performed by using metal measuring dilatometers, equipped with conical covers with a stub for graduated pipes. In 2014, Z. Rusin and P. Świercz [15] proposed a dilatometer in the form of a cylindrical sleeve equipped with a cover with a connector through which the temperature sensor cable was connected. This solution, despite many advantages, did not provide full control over the position of the sensors, and their replacement or change of position was time consuming and forced the discontinuation of the research [15]. Currently, due to the derivation of the temperature sensor, the test sample does not need to be additionally drilled in order to be able to embed the sensor in it. A core borehole of an appropriate diameter and height is sufficient without the need for additional interference with its shape [16]. Freezing of free water in materials, the vast majority of which is essentially free from adhesion forces [17], may take place under conditions of full and/or limited deformation freedom. When analyzing the phase change of water from liquid to solid, the ovules with a structure characteristic of the ice structure play an important role. During the process of cooling the water below phase equilibrium temperature, these seeds can grow to macroscopic sizes of ice crystals. As a result, the greater the value of water supercooling below the phase equilibrium temperature, the faster the freezing process takes place. The kinetics of ice crystal growth Vkr depending on the water supercooling value ΔT is described by equation [17].

V_kr_ = 0.035 × (∆T)^2^ [cm/s] (1)

In the pressure range from 0 to 3000 MPa and temperatures from −120 °C to +100 °C, according to Fletcher [18], there are nine allotropic forms of ice. From a technical and operational point of view, only plain ice is important with hexagonal crystal structure. It occurs at pressures from 0 to about 200 MPa and at temperatures below 0 °C. The density of this ice at atmospheric pressure, and a temperature of 0 °C is 0.917 g/cm^3^ [19]. Free water occurs in relatively large amounts during the freezing process, until the system reaches a critical temperature of −22.4 °C. At lower temperatures, complete solidification of water takes place; however, assuming a constant volume of the system. In fact, free water freezing in large pores of rock material depends on at least two factors:

- Elastic deformability of pore walls;
- The flow of water through capillary pores into voids.

The deformability of the pore walls is practically manifested as an elastic deformation or cracks in the pore space and the formation of a gap into which unfrozen water can move [11]. Thanks to this, it is possible to indirectly determine the size of pores in their largest cross section, as well as the cross sections of the pores connecting them with the entire pore space.

## 2. Materials and Methods

**Figure 1:**
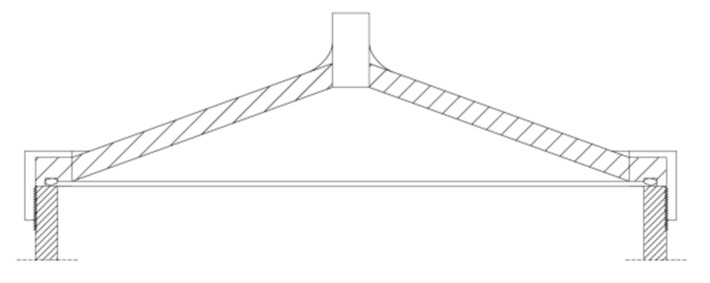
Diagram of the dilatometer cover.

**Figure 2:**
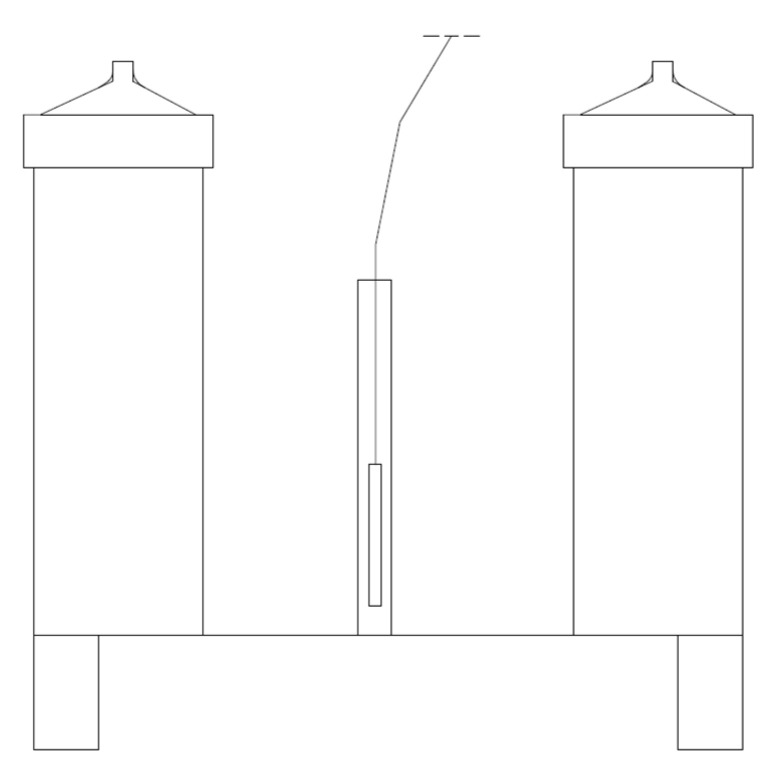
System of measuring dilatometers.

A characteristic element of the dilatometric system was a conical cover with one opening enabling the connection of a graduated measuring tube, as shown in Figure 1. The dilatometric system used in the research is shown in Figure 2.

The measuring capillary connector is attached to the cover by means of a soldered connection. The shape of the cover allowed for easy venting of the dilatometer filled with kerosene [16]. The temperature sensor is located between dilatometers in a special sleeve. The hermetic hydraulic system made it possible to precisely measure changes in the dilatometer volume during cooling and heating.

Measurement dilatometers in Figure 2 were placed on a base equipped with stabilizing feet. A stably mounted sleeve with a temperature sensor was placed between the dilatometers.

**Figure 3:**
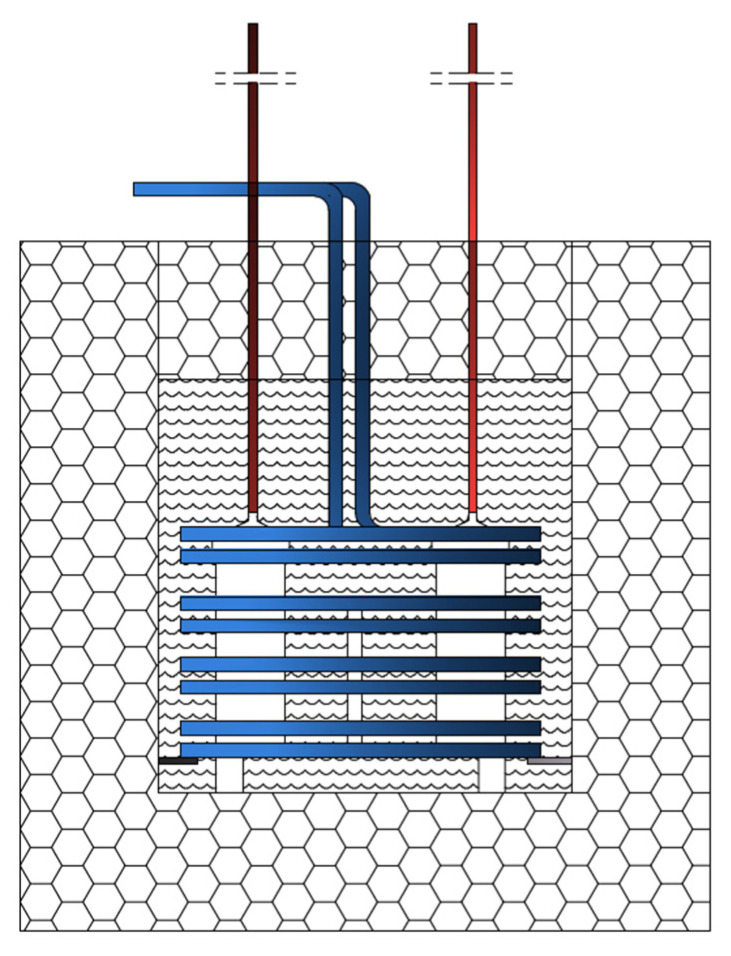
Diagram of an external measuring system [16].

One of the dilatometers contained a water-soaked sample of the tested material, and the other a reference sample soaked in kerosene. With respect to the dilatometers (Figure 3), graduated measuring tubes were attached, and the entire system was housed in a stainless-steel housing. Everything was flooded with kerosene. Dilatometers were placed in an insulated container filled with glycol. Cooling and heating processes of dilatometers were carried out by using a spiral heat exchanger attached to the housing with steel connectors by cooling and heating thermostat filled with glycol. The electronically controlled system made it possible to set the program in any way. On the basis of the performed tests, it was found that the temperature of both dilatometers in each cooling and heating phase was identical [16].

Changes in fluid levels in the measurement tubes, reflecting the volumetric changes of dilatometers with the sample volume, were recorded automatically at 1 min intervals. Then, the level of kerosene was read from the photos with an accuracy of 0.01 cm3. Each time, the measuring dilatometers were set in the same way in order to prevent possible mistakes. The test sample was always on the left, and the reference sample was on the right. The information obtained in this manner was used to compile a summary of changes in volume and the corresponding temperatures in a spreadsheet. The result of the measurement was the value of ∆V’, which was equal to the difference in strains of dilatometer 1 and 2 and was related to time or a specific temperature inside the thermostat chamber:

∆V′ = ∆V_1i_ − ∆V_2i_ (2)

where V_1i_ and V_2i_ are the kerosene levels read at the moment in the measuring tube of dilatometer 1 and 2.

The temperature was also recorded during the entire measurement cycle with the frequency of one minute. Relative volume changes of the samples were recorded with an accuracy of ±0.01 mL, temperature changes with an accuracy of ±0.1 °C and the amount of kerosene in the measuring dilatometers was determined with an accuracy of ±0.1 mL.

On this basis, the mass of ice formed m_L_/V_p_ was calculated in relation to temperature and time.

**Figure 4:**
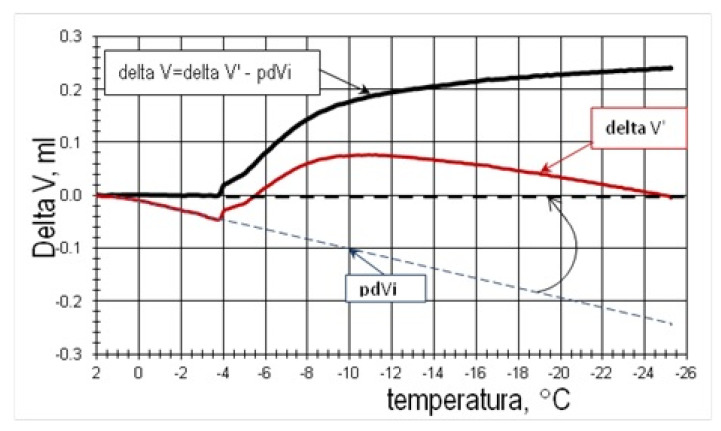
The method of determining pdV_i_ correction and calculating the deformation ΔV [10].

For the analysis of the measurable deformation effects of the phase change of water, it was assumed that the water during soaking with the vacuum method filled all the available pores of the material. Parameter ΔV was analyzed, which, according to the method description, means changes in the volume of the tested sample, resulting from the phase transformation of water (after making appropriate corrections—Figure 4). By knowing sample volume, water content and its porosity, the following was calculated:

- Mass of ice formed at a given temperature—m_L_;
- Deformation effect EO, determining the calculated ice content in proportion to the total water content in the pores;
- Mass of ice per volume unit of rock—m_L_/V_p_;
- The volume of the ice-filled pores at a given temperature (taking into account the adsorbed, unfrozen layer of water directly adjacent to the surface of the pore walls).

An EO value of 100% means that all water in the pores of the material was frozen.

In order to check the accuracy of recorded temperatures, the measuring system was calibrated during the freezing and thawing process. For this purpose, measuring dilatometers containing only the coolant were equipped with temperature sensors. In this system, both dilatometers were equipped inside with electronic sensors that automatically recorded temperature and another one sensor, which was normally located between the dilatometers (according to Figure 2, patent pending). The measuring system prepared in this manner was cooled down to the temperature of −25 °C, and the temperature was briefly stabilized and re-heated to the temperature of +5 °C, with the simultaneous registration of thermometer readings. Temperature values recorded by the measuring system in measuring dilatometers and between them were compared with each other. It was found that the selected cooling speed, the appropriate cooling time of the sample before the start of the measurement and, above all, the introduction of the external system with cooling using a coil allowed minimizing the temperature difference between the system and the inside of the dilatometers. Based on the recorded temperatures, they were recalculated, and corrected values were used for further calculations.

The following assumptions were made:

- The relative deformation of rocks caused by the temperature difference ΔT is negligibly small—the linear expansion coefficient α for rocks ranges from about 3.3 to 38 × 10^−6^;
- It is also many times smaller than kerosene and can be omitted;
- The thermal deformation of pre-freezing water and post-freezing ice is negligible and can be neglected;
- Significant thermal deformability of kerosene causes changes in ΔV caused by a relative change in temperature ΔT.

As the recorded results of deformation changes of samples are significantly influenced by differences in the volume of kerosene in dilatometers and the difference in specific heat of the test and reference sample, an appropriate correction was introduced, which was determined by the graphical method. Then, the base line was plotted as an extension of the ΔV′ value segment in the time immediately preceding the water freezing initiation process—pdV_i_ (Figure 4). Thus, a sequence of ∆V values was obtained that approximated the deformation of the tested material, which is a derivative of the phase change of water.

Currently, the deviation in measurements is small and results mainly from the differences in the volume of kerosene. They are the result of the fact that the comparative sample was soaked with kerosene, while the test sample was soaked with water. The pdV_i_ correction was taken into account in the calculations.

On the basis of the known value of ΔV and the water content in the tested sample mw, it is possible to calculate the ice content in the sample at a given temperature (m_L_) and the EO index, defining the percentage of the amount of freezing water:

m_L_ = ∆V/0.0917 (3)

where m_L_ is the mass of ice (g):

EO = ∆V/(m_w_ × 0.0917) × 100% (4)

where EO is the measured deformation effect (%), m_w_ is the mass of water filling the pores in the sample (g) and ΔV is the relative change in the volume of the frozen sample (cm^3^).

Ice weight (m_L_) is determined by assuming that all open pores are pre-filled with water. Theoretically, it may happen that, after intensive soaking, isolated empty pores will form in the sample. If, as a result of high hydraulic pressures generated during freezing, access to them is blocked, e.g., by destroying the local pore space, part of the water may freeze inside them and then it will not be possible to register this fact by using ΔV measurements. Consequently, deformation is more carefully interpreted as the lower limit of the possible phase transformation of water to ice.

The second important and very useful analysis index in the DAVS method is the calculated water content undergoing water–ice phase change per unit volume of the tested material: m_L_/V_p_.

An important issue in calculating the actual pore volume is the amount of water that is adsorbed on the inner pore surfaces and that does not undergo phase change. Therefore, the value of such a layer should be added to the calculated volume of ice formed in the sample. When we are dealing with pores with small diameters—on the order of a few nanometers—the volume of such a layer is a relatively large part of the volume of the pore. As a result, in order to be able to determine pore size distribution in the tested material as accurately as possible, the thickness of this layer was assumed to be 0.8 nm in accordance with the literature [20]. It was also assumed that it is constant in the processes of cooling and heating the material. Another assumption was the adoption of a cylindrical pore shape (based on the Gibbs–Thomson equation) in the entire system [21]. Moreover, it was assumed that the water contained in the material did not move to the surface of the sample or to empty, isolated pores during solidification. It was also assumed that the impact of cracks caused by water freezing on the pore space is insignificant [22].

## 3. Results

A dozen of samples of carbonate rocks (limestones and dolostones) from domestic deposits were examined by the method of differential analysis of volumetric strain.

By analyzing the results of measuring the full hysteresis of m_L_/V_p_, a significant, disturbing effect of the thermal inertia of the system and the intensity of the phase change of water on the possibility of precise assignment of certain phenomena to the actual temperatures at which they occur was found. Leaving such registered effects without introducing corrections results in erroneous calculation of the actual pore radius size distribution. There are known methods to limit the impact of this phenomenon on the precision of calculations used in calorimetric tests. In the case of the DAVS method, the method of elimination or partial elimination of this unfavorable phenomenon is different [23,24]. One method to reduce the effect of system inertia is to reduce the rate of cooling and heating of the material. Nevertheless, even with a small rate of cooling and heating the sample, there will be a certain temperature shift value of the recorded deformation effects in relation to the temperatures at which they actually occur. This is a problem not only of the thermal inertia of the system but also of exogenous and endothermic during freezing and thawing of water contained in the pores of the sample. Relatively large masses of water in the pores may impose additional thermal effects on the inertial effect, delaying the registration of phase change phenomena in relation to the temperature of the cooling or heating medium, measuring dilatometers. The first collision with this problem occurs with the initiation of water nucleation as it cools. Two phenomena accumulated: supercooling of water compared to the theoretical freezing start temperature and triggering in a short time; and a large dose of phase transformation energy, significantly slowing down the process of cooling the inside of the sample and continuing freezing. The system begins to “catch” the equilibrium at relatively low temperatures, which makes it impossible to analyze the phenomena occurring at higher temperatures, including the calculation of the pore size in a fairly large range of their dimensions. The second time is when there is a significant disturbance of the relatively linear relationship: the temperature of the heating medium and the temperature of the inside of the sample, which is observed during water defrosting at temperatures above −5 °C. This mainly applies to materials with a lot of pores and large pores.

In the version of the measuring system used a few years earlier [10,13], the significance of the problem of thermal inertia was limited, because one of the temperature sensors was inside the sample, which allowed for constant monitoring of the temperature inside the material. In the discussed tests, the internal sensor was not used because it caused major technical problems, starting with the difficulty of drilling a hole in the sample without damaging it and the lack of tightness of the measuring system. The development of an appropriate correction, necessary for a significant reduction in the discussed disturbances in the current measurements, consisted in the analysis of the rock tests performed earlier [25], where the internal temperature of the samples was recorded. The method of converting the m_L_/V_p_ chart and a description of the assumptions for this process are presented below.

The temperature inside the sample was estimated on the basis of the results of the studies described in the aforementioned publications and reports [25,26], where the same type of material was used, coming from geologically identical deposits. The water absorption of the material was also taken into account. Efforts were made to ensure that the water content in the reference samples was similar to that contained in the research material. The method of capillary soaking of the compared materials was also analyzed. In this manner, for almost every material, a reference sample was available from previous tests that used a temperature sensor inside the sample, which allowed for an approximation of actual temperature values. Knowing the temperatures inside the material and assuming that we only convert some of the results from the endothermic process, for temperatures above −5 °C, the temperature inside the thermostat chamber was changed in the calculation algorithm to the temperature of the sample interior. The temperature of −5 °C has been defined as the limit temperature in this conversion; however, the differences in temperature in the further part of the measurements between the inside of the sample and the inside of the thermostat were not large. Thus, the temperature change in the further part of the endothermic process did not cause discontinuities and disturbances in the graph that would create additional complications. In this manner, the values for all samples used in the DAVS tests were recalculated.

**Figure 5:**
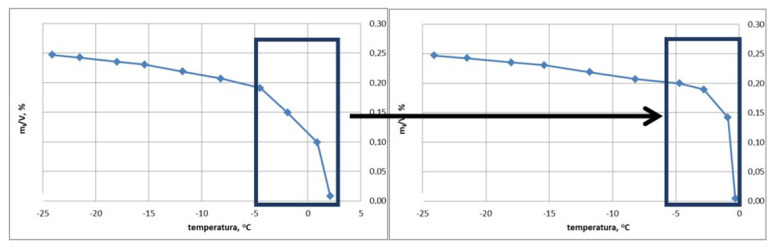
Example of changing the m_L_/V_p_ plot in a sample during the heating process.

Figure 5 shows an example of the m_L_/V_p_ graph during the heating process, which was converted according to the analysis of the temperature value of the reference sample from previous tests (temperature sensor inside the sample).

The author is aware of a certain flaw in the proposed correction; however, the adopted method enables further analyses to be carried out with less error compared to the analysis of the original recorded results.

Bearing in mind that the initial stage of water freezing due to the specificity of the water crystallization process, especially in isolated pores (connected by pores with much smaller cross-section sizes) and the random and spontaneous nature of the nucleation onset, made it impossible to calculate the internal dimensions of the pores, the effects recorded during the ice melting phase were used to analyze the volume and dimensions of the pores [21].

Table 1 shows ice content at individual temperatures, m_L_/V_p_, during the exothermic and endothermic process after taking into account appropriate corrections for selected rock samples.

In all analyzed cases, the amount of ice material formed in the pores increases as the temperature of the system decreases, starting from different values at a temperature of −5 °C, considered conventionally as the beginning of the thermodynamically balanced freezing phase (end of the spontaneous nucleation phase).

In turn, when heating, it is easy to see that the melting process in most cases in the temperature ranging from −25 °C to −5 °C had little or no occurrence (B1, F1, F3, F4 and H3). All or most of the ice thawing process occurred at temperatures higher than −5 °C. The differences in the behavior of individual rocks obviously depend on the size of the internal pores and their connections.

**Figure 6:**
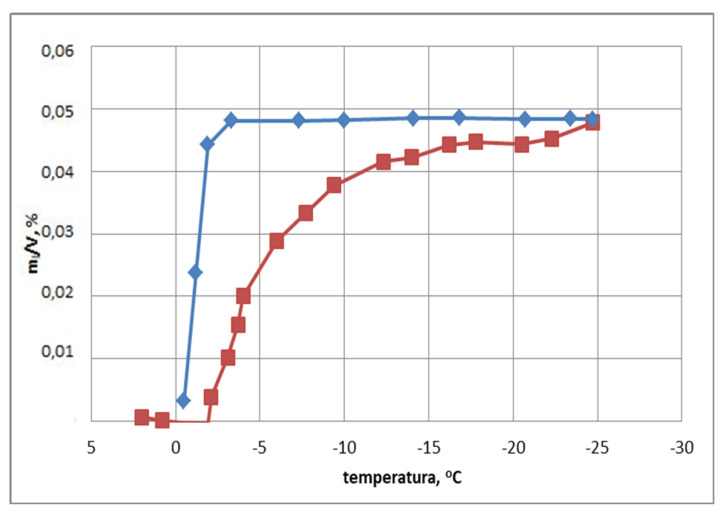
Full hysteresis of m_L_/V_p_ in sample B1.

The plot of full hysteresis, obtained on the basis of tests by the method of differential analysis of volumetric strain, is shown in Figure 6 (for an exemplary sample B1).

The example of sample B1 shows that, from the very beginning, the water in the sample freezes intensively as the temperature drops. Ice thaws above −5 °C. This proves the presence of small and large diameter pores in the sample, as well as bottle-shaped pores, of which there is a significant number.

**Figure 7:**
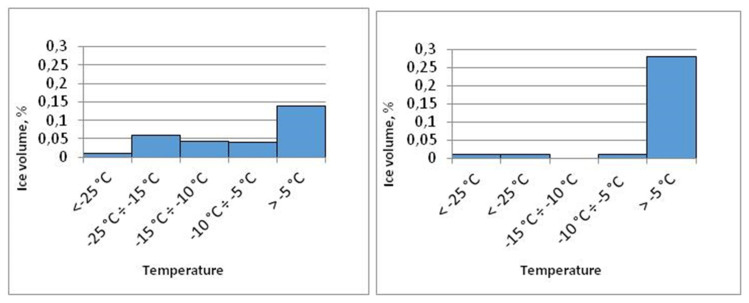
Distribution of ice volume in sample B1—during cooling (**left**) and heating (**right**).

On the basis of the m_L_/V_p_ value and the temperature, it is possible to calculate (using Bruno’s [27] rule) the pore volume occupied by ice in the following phase transition temperature ranges: <−25 °C; −25 °C ÷ −15 °C; −15 °C ÷ −10 °C; −10 °C ÷ −5 °C; and >−5 °C. Figure 7 shows a diagram of the pore volume occupied by the ice of the selected sample (B1). It is worth noting that pore volume is calculated as the sum of the volume of ice and the non-frozen layer of adsorbed water. The relative proportion of adsorbed water to ice volume increases as pore size decreases.

Pores with radii > 0.02 µm are the dominant group of pores in the tested material. The graph of the ice volume distribution during the cooling and heating process shows a certain bottle-shaped pore volume. This is evidenced by the significant total volume of pores occupied by ice at lower temperatures, which range from −25 °C to −5 °C. This temperature range corresponds to the pore junction radii of 0.003 ÷ 0.02 µm.

It is obvious that by using the DAVS method it is possible to investigate the size of the pores only when there is a measurable deformation effect of the phase change of water or ice in them. In the case of empty pores (not containing water), it is impossible to determine their size distribution using the described method. Additionally, the mass of ice in the bottle pores for individual temperature ranges was calculated from the formula:

m_LB_ = m_LZT_ − m_LTR_ (5)

where m_LB_ is the mass of ice in the bottle pores; m_LTZ_ is the ice mass in a specific temperature range when frozen; and m_LTR_ is the mass of ice in the same temperature range during thawing.

The remaining mass of ice that melts in the same temperature range, determined by the endothermic process, can be related to pore size. In this case, their calculated volume also contains adsorbed water. These pores refer to pores connecting the bottle-shaped pores and other small pores of the same or similar size. An additional assumption allowing the correct calculation of the volume of the bottle-shaped pores was that their dimensions were greater than the cross-sectional value corresponding to the temperature of −5 °C. The calculated mass of ice in the bottle pores was finally assigned to pores with radius dimensions > 0.02 µm. In determining pore volume, the volume of the water film adsorbed on the inner surfaces of the 0.8 nm thick pores was taken into account.

## 4. Conclusions

On the basis of the research results obtained by the DAVS method, the size of the pores in the material (mesopores) and the connections between them can be estimated. Additionally, it is possible to determine the pore volume in the individual ranges of their radii. Moreover, by interpreting the results, one can obtain the pore volume occupied by water, which freezes down to −25 °C. The volume of pores occupied by water that does not freeze, and you can also determine the volume of pores that are inaccessible to water. As part of the research, it was important to determine the full hysteresis of changes in the sample volume: freezing–thawing.

The improved DAVS research method allows for the registration of the full hysteresis of the water–ice–water phase change. The new system of measuring dilatometers has been registered as a patent in the Patent Office of the Republic of Poland under number 432844. In the DAVS method, by analyzing the charts of freezing rock samples, it was possible to determine the size of the entries into the bottle-shaped pores. Based on the heating diagrams, the dimensions of the pores themselves were determined similarly to the use of low-temperature DSC calorimetry. It was also possible to estimate the volume of the bottle-shaped pores as well as the entrances leading thereto. Using the DAVS method, it was also possible to estimate pore volumes that fill with water that were unable to phase change. The pore volumes calculated in the DAVS method can be assigned specific ranges of their radii values. It was not possible to separate smaller pores from larger pores above a radius of 0.02 µm.

## 5. Patents

432844—The new system of measuring dilatometers registered as a patent in the Patent Office of the Republic of Poland; authors: K. Skowera and Z. Rusin.

## Figures and Tables

**Table 1:** List of m_L_/V_p_ ratios determined by the DAVS method during freezing and thawing process.

No	m_L_/V_p_ (−5 °C)	m_L_/V_p_ (−10 °C)	m_L_/V_p_ (−15 °C)	m_L_/V_p_ (−25 °C)	m_L_/V_p_ (−15 °C)	m_L_/V_p_ (−10 °C)	m_L_/V_p_ (−5 °C)
A1	0.020	0.032	0.032	0.034	0.032	0.031	0.026
A2	0.016	0.017	0.017	0.029	0.026	0.026	0.022
A3	0.023	0.025	0.030	0.036	0.035	0.033	0.031
B1	0.024	0.038	0.042	0.048	0.048	0.048	0.048
B2	0.007	0.010	0.019	0.041	0.039	0.036	0.028
B3	0.013	0.016	0.018	0.023	0.022	0.022	0.018
C1	0.005	0.010	0.017	0.057	0.055	0.052	0.047
C2	0.010	0.013	0.019	0.024	0.023	0.022	0.020
C3	0.017	0.027	0.029	0.038	0.037	0.033	0.030

## Data Availability

The data presented in this study are available on request from the corresponding author.
